# *Corynebacterium oculi*-related bacterium may act as a pathogen and carrier of antimicrobial resistance genes in dogs: a case report

**DOI:** 10.1186/s12917-023-03821-y

**Published:** 2023-11-29

**Authors:** Milena Tresch, Christine Watté, Michele Stengard, Corinne Ritter, Isabelle Brodard, Simon Feyer, Estelle Gohl, Ezgi Akdesir, Vincent Perreten, Sonja Kittl

**Affiliations:** 1 Institute of Veterinary Bacteriology, Vetsuisse Faculty, University of Bern, Bern, Switzerland; 2 Department of Clinical Veterinary Medicine, Vetsuisse Faculty, University of Bern, Bern, Switzerland; 3 Cabinet Vétérinaire Villereuse, Geneva, Switzerland

**Keywords:** *Corynebacterium oculi*, MLS_B_, Fluoroquinolones, Keratitis, Cystitis

## Abstract

**Background:**

The genus *Corynebacterium* comprises well-known animal and human pathogens as well as commensals of skin and mucous membranes. Species formerly regarded as contaminants are increasingly being recognized as opportunistic pathogens. *Corynebacterium oculi* has recently been described as a human ocular pathogen but has so far not been reported in dogs.

**Case presentation:**

Here we present two cases of infection with a novel *Corynebacterium sp.*, a corneal ulcer and a case of bacteriuria. The two bacterial isolates could not be identified by MALDI-TOF MS. While 16 S rRNA gene (99.3% similarity) and *rpoB* (96.6% identity) sequencing led to the preliminary identification of the isolates as *Corynebacterium* (*C.*) *oculi*, whole genome sequencing revealed the strains to be closely related to, but in a separate cluster from *C. oculi*. Antimicrobial susceptibility testing showed high minimal inhibitory concentrations of lincosamides, macrolides, tetracycline, and fluoroquinolones for one of the isolates, which also contained an *erm*(X) and *tet*-carrying plasmid as well as a nonsynonymous mutation leading to an S84I substitution in the quinolone resistance determining region of GyrA.

**Conclusions:**

While the clinical signs of both dogs were alleviated by antimicrobial treatment, the clinical significance of these isolates remains to be proven. However, considering its close relation with *C. oculi*, a known pathogen in humans, pathogenic potential of this species is not unlikely. Furthermore, these bacteria may act as reservoir for antimicrobial resistance genes also in a One Health context since one strain carried a multidrug resistance plasmid related to pNG3 of *C. diphtheriae*.

**Supplementary Information:**

The online version contains supplementary material available at 10.1186/s12917-023-03821-y.

## Background

Corynebacteria are Gram-positive club-shaped bacteria belonging to the family of the *Corynebacteriaceae* and the order of the *Mycobacteriales*. The genus *Corynebacterium* (*C.*) currently consists of 141 recognized species (https://lpsn.dsmz.de/genus/corynebacterium, accessed 03.03.2023), isolated from a large variety of host species as well as environmental sources. The genus contains some well-known pathogens such as *C. diphtheriae* causing diphtheria in humans and *C. pseudotuberculosis* causing pseudotuberculosis in goats and sheep (a notifiable disease in Switzerland). *C. pseudotuberculosis* as well as *C. ulcerans* can encode the diphtheria toxin and cause zoonotic infections [1]. Nevertheless, many corynebacteria were usually considered as incidental findings rather than pathogens in the past. The role of corynebacteria found in clinical samples can be difficult to determine as they occur as commensals in various body sites including the mucosal surfaces. More recently, increasing numbers of *Corynebacterium* species are being recognized as opportunistic pathogens [2]. In dogs, corynebacteria are considered normal inhabitants of healthy skin, however, *C. auriscanis* and other species seem to occur more frequently in inflamed ears than healthy ears. But as they are almost always isolated as a mixed culture from cases with otitis externa, their contribution to the condition is still unclear [3]. *C. provencense* has been reported as a cause of otitis in a cat [4], and *C. urealyticum*, known to be involved in the pathogenesis of encrusted cystitis and encrusted pyelitis in humans, has also been described as a rare cause of cystitis in dogs [5]. Several *Corynebacterium* species have been isolated both from the conjunctival sacs of healthy dogs and from dogs with corneal ulcers [6]. In recent years, corynebacteria are increasingly recognized as ocular pathogens in humans, associated with keratitis, corneal ulcers and conjunctivitis [7]. Corynebacteria associated with human ocular infections, previously classified as *C. mastitidis*, were found to belong to two novel species, namely *C. oculi* and *C. lowii* [8].To date, neither of these species have been reported in dogs or cats.

Corynebacterial infections can be problematic due to the potential of developing antimicrobial resistance, especially to quinolones, which are frequently used in both human and veterinary medicine [4, 9, 10]. Resistance to quinolones is usually due to non-synonymous mutations in the quinolone resistance determining region (QRDR) of the *gyrA* gene [10, 11]. Resistance to lincosamides, macrolides and streptogramin B (MLS_B_) is also known to occur in various *Corynebacterium* species including *C. diphtheriae* and is typically conferred by a 23 S rRNA methyltransferase encoded by the *erm*(X) gene [12–14]. The *erm*(X) gene is located on mobile genetic elements, e.g. plasmids, which can potentially be transferred between strains or even species [12, 15].

Here we report on two infections in dogs with *C. oculi*-related bacteria which have not been previously described. We used cultural and whole-genome approaches to characterize the two strains, one of which showed resistance to quinolones, tetracycline, lincosamides and macrolides.

## Case presentation

Strain 21KM1197 was obtained from an ocular swab of an 11-year-old female Chihuahua with keratitis. The dog was presented with a unilateral anterior stromal ulceration in the ventromedial corneal quadrant. No antimicrobial treatment had been administered before sampling. Corneal samples were taken using a sterile cotton swab and cultured on Trypticase™ Soy Agar II with 5% Sheep Blood (TSA-SB) (BD™) with a *Staphylococcus sciuri* (strain 09KM1127) streak (providing nicotinamide adenine dinucleotide) and on Pasteurella Selective Agar (Thermo Fisher) under 5% CO_2_ atmosphere at 37 °C, as well as on MacConkey Agar No. 3 (Thermo Fisher) aerobically at 37 °C. Roughly 60 grey pinpoint colonies were seen on TSA-SB after 24 h of incubation. No growth occurred on the other media used after 48 h of incubation. Gram-staining revealed short, rod-shaped or almost coccoid Gram-positive bacteria. Identification of the colonies was attempted by MALDI-TOF MS (Bruker, MBT 8468 MSP Library) and VITEK® 2 Compact (CBC, Biomérieux) but yielded no result.

Strain 22KM0430 was obtained from a spontaneous urine sample of a female dog of unknown breed with suspected cystitis. The 14-year-old dog was presented to the clinician because of polyuria and polydipsia, which could not be explained by the results of blood chemistry and urine analysis. No signs of dysuria were observed. The dog had a history of recurring otitis externa but was otherwise healthy and currently not under systemic antibiotic treatment. Ten microliters of urine were routinely cultured aerobically on TSA-SB and ChromAX Urine agar (Axonlab) at 37 °C. Approximately 100 grey pinpoint colonies (amounting to ~ 10^4^ c.f.u. per ml of urine) appeared on TSA-SB after 24 h incubation, together with 37 hemolytic colonies. Resulting colonies were submitted to MALDI-TOF MS identification as described above. The hemolytic colonies were identified using MALDI-TOF MS (Bruker, MBT 8468 MSP Library) as *Streptococcus* (*S.*) *canis*, while the pinpoint colonies could not be identified. The bacteria had the same morphology in Gram-staining as strain 21KM1197.

Since both strains could not be identified using standard methods, marker gene-based characterization was attempted. Approximately five colonies taken from a TSA-SB agar plate were lysed in 100 µl lysis buffer (60mM NaHCO_3_, 50mM Tris, 250mM KCl) for 10 min at 97 °C. This suspension was then mixed with 100 µl of 2% bovine serum albumin and centrifuged for 1 min at 25,000 *x g* and the supernatant was used as a template for PCR. PCR targeting the 16 S rRNA gene was performed using universal primers [16]. Additionally, for strain 21KM1197 a highly polymorphic region of the *rpoB* gene was amplified by PCR as described elsewhere [17]. PCR products were sent to a commercial provider (Microsynth) for Sanger sequencing. Sequences were analyzed with EzBioCloud 16 S rRNA database [18] and NCBI BLAST (https://blast.ncbi.nlm.nih.gov/Blast.cgi). The obtained 16 S rRNA gene sequences of both strains were identical. EzBioCloud analysis of the 16 S rRNA gene partial sequence showed 99.3% similarity with *C. oculi* (GenBank acc. no. KJ938709) and 98.3% similarity with *C. mastitidis* (Y09806). The threshold for species delineation based on sequence identity of the 16 S rRNA gene is 98.65% according to Kim et al. [19]. The partial *rpoB* gene sequence obtained from strain 21KM1197 showed 96.6% identity with *C. oculi* (KJ938694.1) and 93.6% identity with *C. mastitidis* (AY492281.1). The suggested nucleotide identity cutoff value for species differentiation in corynebacteria based on this partial sequence of the *rpoB* gene proposed by Khamis et al. [17] is 96.6%, meaning our strain lies just at the cut-off point.

Therefore, whole genome sequencing (WGS) was performed to further characterize the strains and clarify their taxonomic position. Strains were grown for 48 h at 37 °C in 5% CO_2_ atmosphere on TSA-SB. DNA was extracted using a guanidium thiocyanate and phenol chloroform extraction protocol [20]. For short-read sequencing, a DNA library was prepared using the Illumina® DNA Prep (M) Tagmentation Kit and an IDT® for Illumina® DNA/RNA UD Indexes Set according to the manufacturer’s instructions (Illumina®) and was sequenced at the next generation sequencing platform of the University of Bern, Switzerland. Long-read sequencing was performed using a MinION R.9.4.1 flow cell (Oxford Nanopore Technologies, ONT). Hybrid assemblies of the short and long reads were obtained using Unicycler v. 0.5.0 [21]. The assemblies were annotated using PGAP. The ONT and Illumina® raw read containing files as well as the assemblies of both isolates were deposited into the Sequence Read Archive (SRA) and GenBank of NCBI (BioProject ID: PRJNA892875). For verification of the genome assembly according to Chun et al. [22], the sequences of the 16 S rRNA genes retrieved from the whole genomic data were compared to the sequences resulting from Sanger sequencing and confirmed to be identical. Genome assemblies generated one chromosome and one plasmid for each strain (Table 1). The coverage resulting from the Illumina reads was 600 X for isolate 21KM1197 and 266 X for 22KM0430. Coverage values for the Nanopore reads were 286 X and 296 X for the two isolates, respectively. The obtained genome size of each of the two isolates was 2.386 Mb, which is similar to the genome size of *C. oculi* (NZ_LKST00000000.1) with 2.41 Mb. Isolate 22KM0430 carried a circular 35-kb plasmid (p22KM0430). A partly similar plasmid segment (p22KM1197) which could not be circularized was also detected in isolate 21KM1197 (Table 1).

**Table 1:** Genome assembly information of strains 21KM1197 and 22KM0430

Strain	Genomic location	Number of contigs	Circular	Total length	GC%	Accession
21KM1197	chromosome	1	yes	2,386,360	64.5	CP123907
p21KM1197	plasmid	1	no	18,457	51.0	CP123908
22KM0430	chromosome	1	yes	2,386,539	64.5	CP123905
p22KM0430	plasmid	1	yes	35,562	56.9	CP123906

**Fig. 1:**
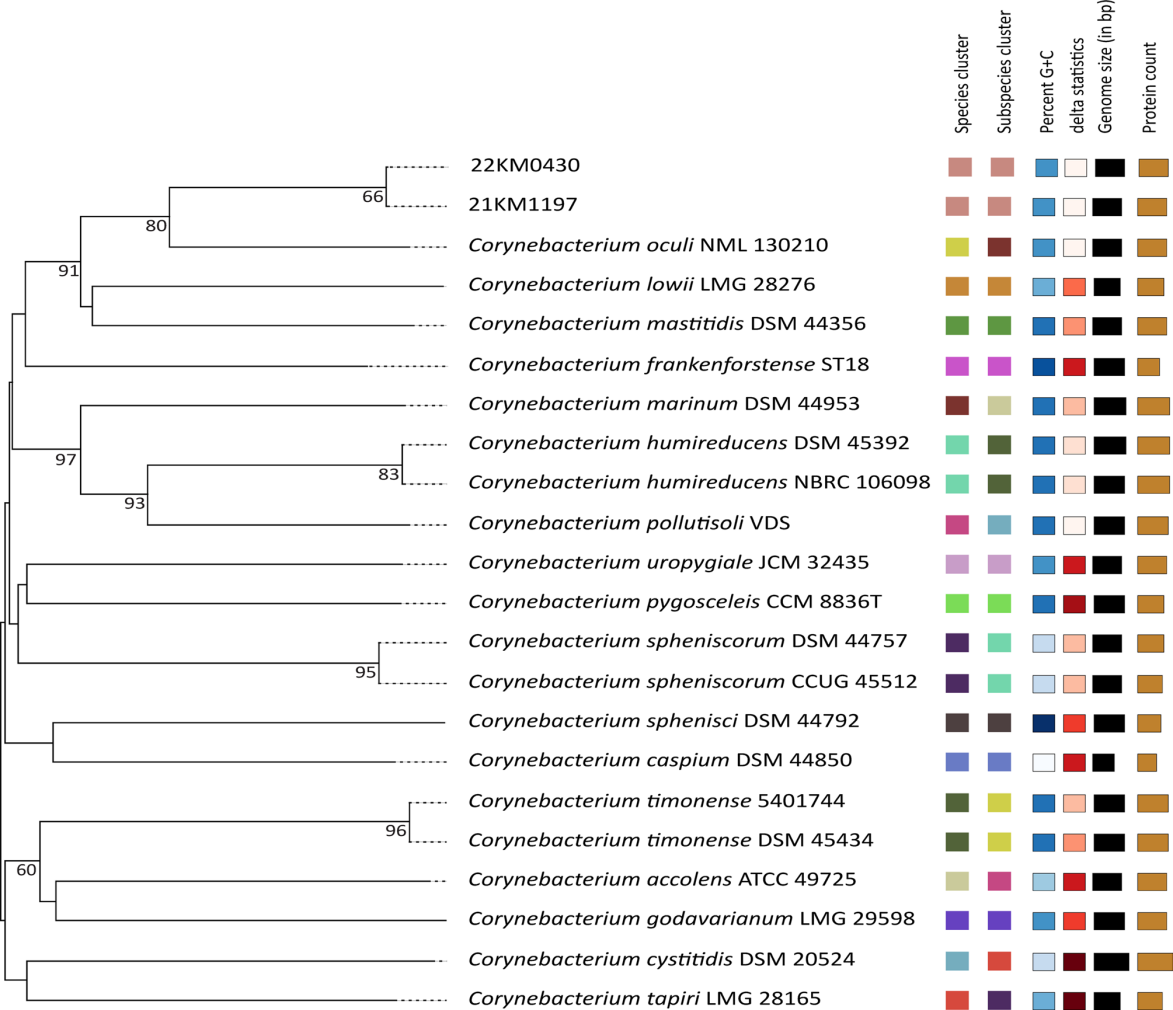
Genome based distance phylogeny (GBDP) as calculated by the TYGS web server. The numbers above branches indicate GBDP pseudo-bootstrap support values > 60% from 100 replications. The tree was rooted at midpoint

For phylogenetic characterization, digital DNA-DNA hybridization (dDDH) and Genome Blast Distance Phylogeny (GBDP) analysis were calculated using the Type Strain Genome Server (TYGS) pipeline [23]. The dDDH value was obtained using formula *d*_*6*_ (sum of all identities found in high scoring segment pairs divided by total genome length) which corresponds best with other phylogenetic methods [24]. The resulting genome-based tree placed strains 21KM1197 and 22KM0430 onto a new branch, which was next to but separated from the branch of *C. oculi* (Fig. 1). A dDDH value of 100% [95% confidence interval (C.I.) 99.9–100] was obtained between strains 21KM1197 and 22KM0430, indicating that they belong to the same species. The dDDH values obtained by comparison with *C. oculi* were 67.1% [C.I. 63.7–70.3] for 22KM0430 and 67.3% [C.I. 63.9–70.5] for 21KM1197 which is below the cut-off of 70% set for species delineation [24]. The dDDH values obtained with *C. mastitidis* were lower with 43.9% [C.I. 40.9–46.9] and 44.0% [C.I. 41.0–47.0] for 22KM0430 and 21KM1197, respectively.

**Fig. 2:**
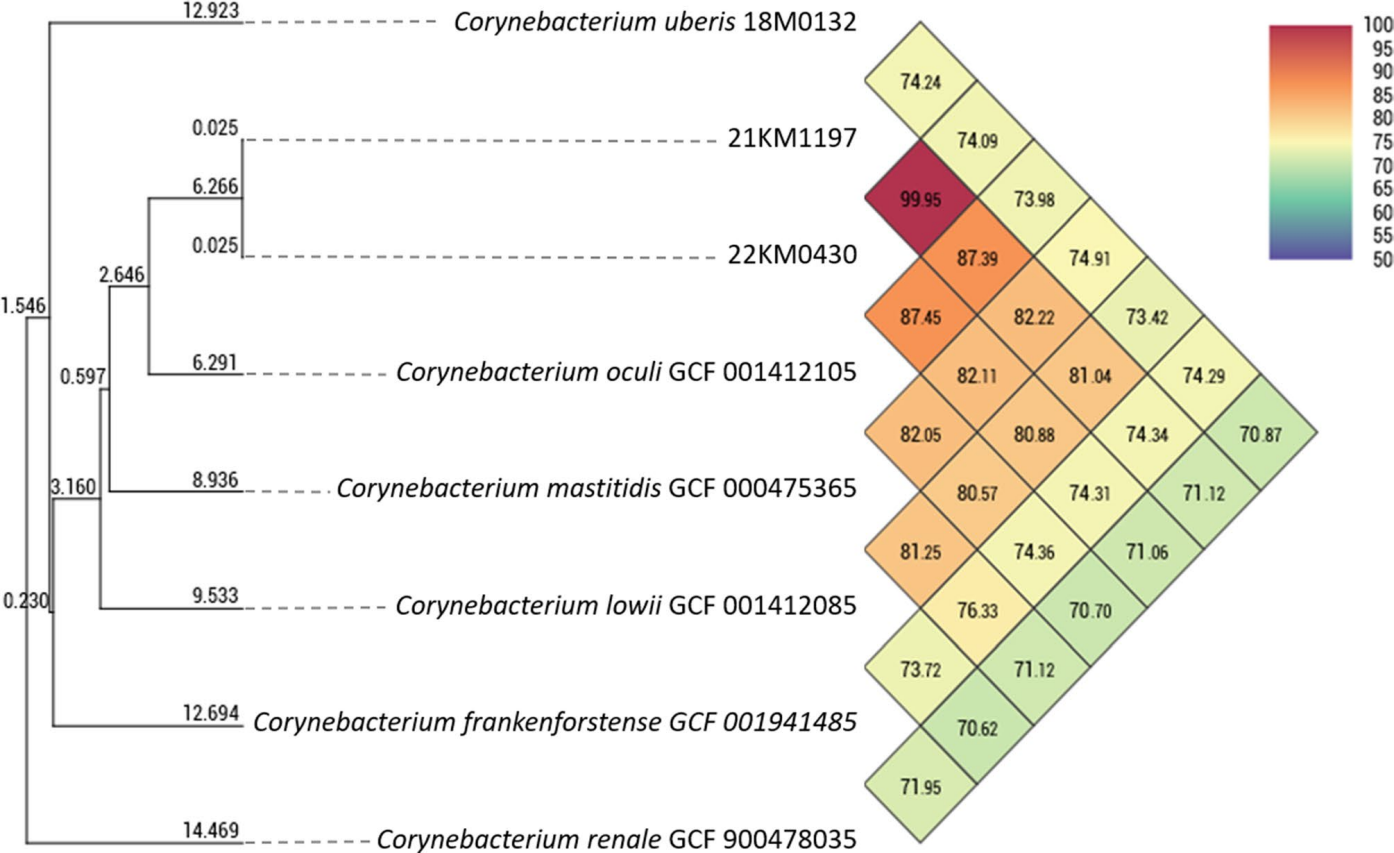
Heat map and corresponding tree generated with OrthoANI values calculated by OAT software

**Fig. 3:**
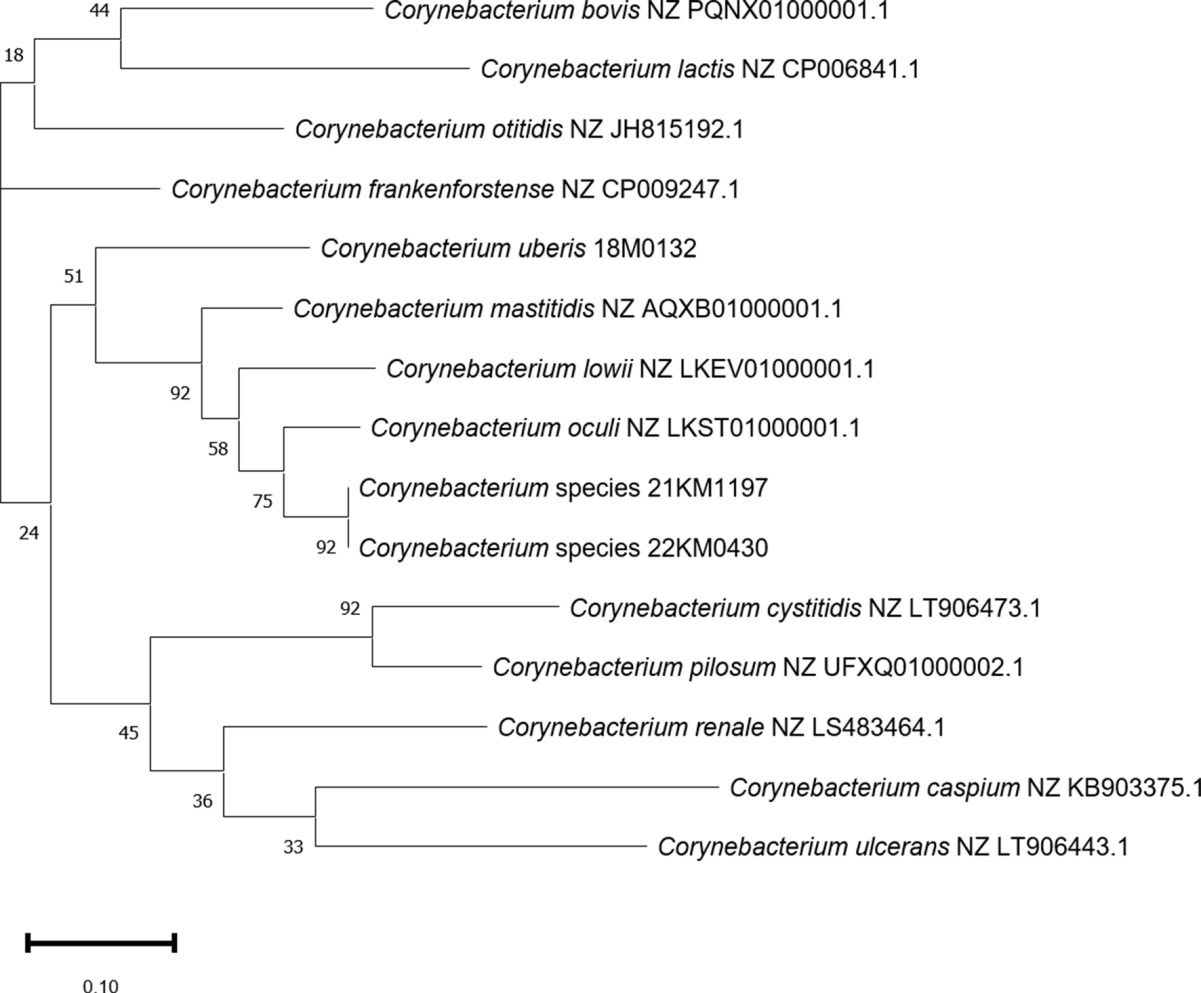
Maximum Likelihood tree created using UBCG. This tree is based on 92 bacterial core genes. Branch numbers indicate the number of gene trees that support this branching (maximum 92)

Average nucleotide identity (ANI) was determined using the OrthoANI Tool (OAT) v0.93.1 [25]. ANI generated similar identification results to dDDH but showed a clearer separation of 21KM1197 and 22KM0430 from *C. oculi*. The two strains shared an ANI of 99.95% whereas ANI was only 87.39% and 87.45% with the type strain of *C. oculi*, which is the most closely related species. The ANI-based species delineation is situated at around 95% [26] indicating that the two strains presented here likely belong to a novel, previously undescribed species. A phylogenetic tree and a heat map inferred from the ANI values are shown in Fig. 2. Phylogeny based on the bacterial core genome was analyzed using UBCG [27]. This phylogeny, which is based on 92 bacterial core genes, also placed both 22KM0430 and 21KM1197 close to *C. oculi* but in a separate branch (Fig. 3). Together the whole genome based phylogenetic analyses indicate that strains 21KM1197 and 22KM0430 belong to a novel, previously undescribed species.

Antibiotic susceptibility testing was performed for both strains using the broth microdilution method according to CLSI standards with custom Sensititre® plates NLD1VMON, NLD3VMON and NLD4VMON (Thermo Scientific™). Sensititre® plate batches were tested with control strains *E. coli* ATCC 25922 (NLD1VMON and NLD3VMON), and *S. pneumoniae* ATCC 49619 (NLD4VMON) which yielded expected results. In brief, corynebacteria were suspended in cation adjusted Mueller-Hinton broth with TES (Sensititre®, Thermo Scientific™) containing 4.95% lysed horse blood to obtain an inoculum of approximately 5 × 10^5^ colony forming units (c.f.u.) ml^− 1^. Plates were read after 48 h aerobic incubation at 36 °C. Minimal inhibitory concentration (MIC) values were interpreted according to breakpoints from CLSI (M45Ed3E) where available. The MIC values of strain 21KM1197 (keratitis) and 22KM0430 (cystitis) are shown in Table 2. Isolate 21KM1197 showed low-range MIC values for all tested antibiotics, which were interpreted as susceptible. Isolate 22KM0430 showed resistance to clindamycin, erythromycin, ciprofloxacin and tetracycline and high-range MICs for enrofloxacin, marbofloxacin, tilmicosin and tulathromycin for which no breakpoints are available.

**Table 2:** MIC values for isolates 21KM1197 and 22KM0430 from keratitis and cystitis, respectively. High-range MIC values are marked in bold. Interpretation is according to breakpoints from CLSI (M45Ed3E) where available. Detected resistance mechanisms are indicated for strain 22KM0430 in a separate column

Antibiotic	Range tested [mg/L]	Interpretation and MIC 21KM1197		Interpretation, mechanism and MIC 22KM0430			Resistance breakpoint [mg/L]
Ampicillin	0.03-64		0.12			2	
Amoxicillin/clavulanic acid 2:1	0.03/0.015–64/32		0.12/0.06			1/0.5	
Penicillin	0.015-32	S	0.12	I		2	≥ 4
Ceftiofur	0.03-64		1			2	
Cefquinome	0.015-32		0.12			0.5	
Cephalotin	0.015-128		<= 0.06			0.25	
Cefotaxime	0.015-32	S	0.5	S		1	≥ 4
Cefoperazone	0.06-32		2			4	
Ciprofloxacin	0.008-16	S	0.12	R	*gyrA* p.S84I	**> 16**	≥ 4
Clindamycin	0.03-64	S	0.5	R	*erm*(X)	**> 64**	≥ 4
Gentamicin	0.12–256	S	<=0.12	S		<=0.12	≥ 16
Enrofloxacin	0.008-16		0.12		*gyrA* p.S84I	**> 16**	
Erythromycin	0.015-32	S	0.06	R	*erm*(X)	**> 32**	≥ 2
Trimethoprim/Sulfamethoxazole	0.015/0.29–32/608	S	0.25/4.75	S		0.25/4.75	≥ 2/38
Marbofloxacin	0.008-16		0.25		*gyrA* p.S84I	**> 16**	
Tetracycline	0.12–256	S	1	R	*tet*	**64**	≥ 16
Tilmicosin	0.06–128		2		*erm*(X)	**> 128**	
Tulathromycin	0.06-32		0.5		*erm*(X)	**> 32**	

**Fig. 4:**
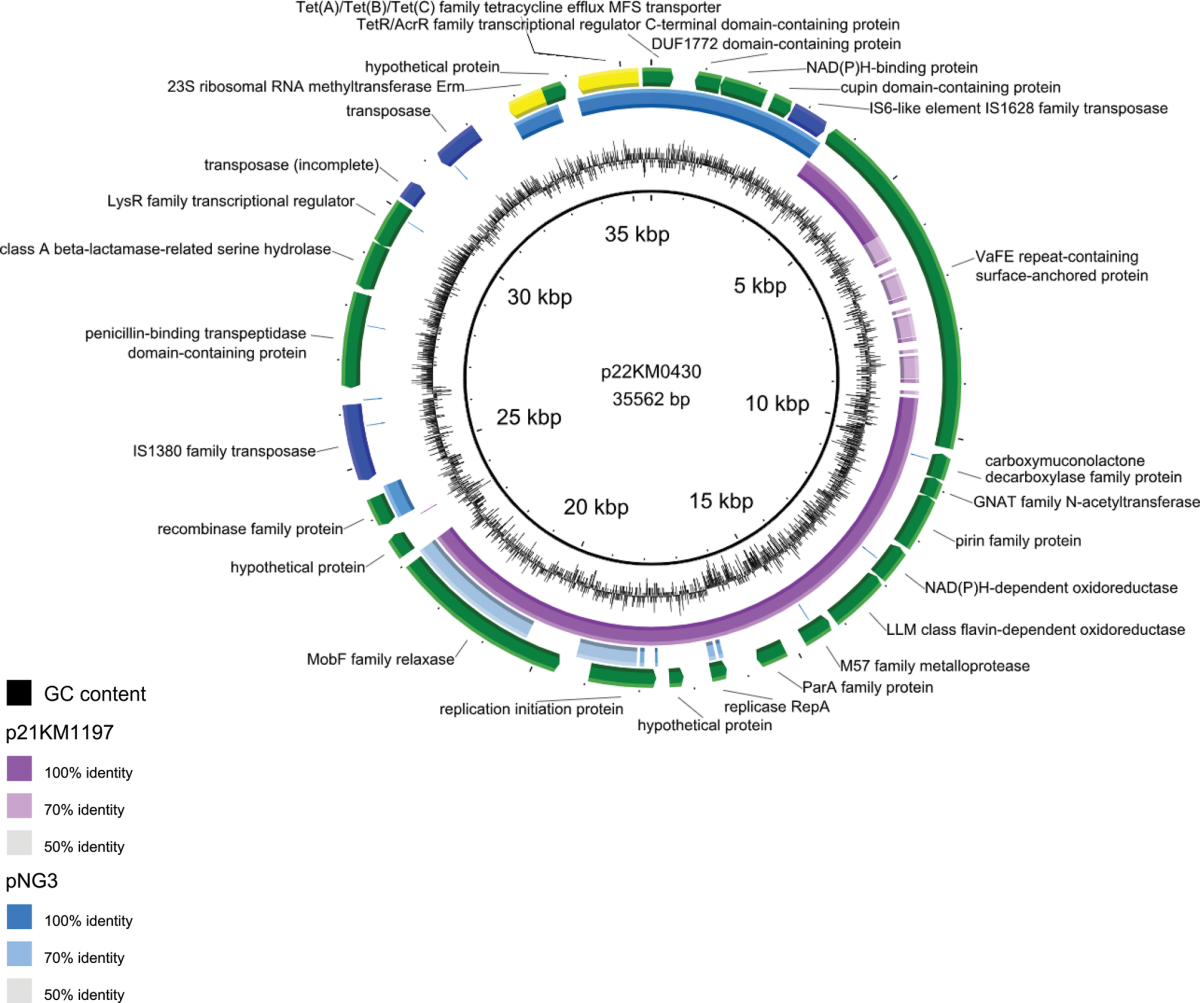
BLASTn alignment of plasmid p22KM0430 of *Corynebacterium* sp. strain 22KM0430 (inner ring) with the plasmid p21KM1197 found in *Corynebacterium* sp. strain 21KM1197 and the plasmid pNG3 (GenBank Accession: MZ348427.1). Figure generated using BLAST ring generator (BRIG [37])

**Fig. 5:**
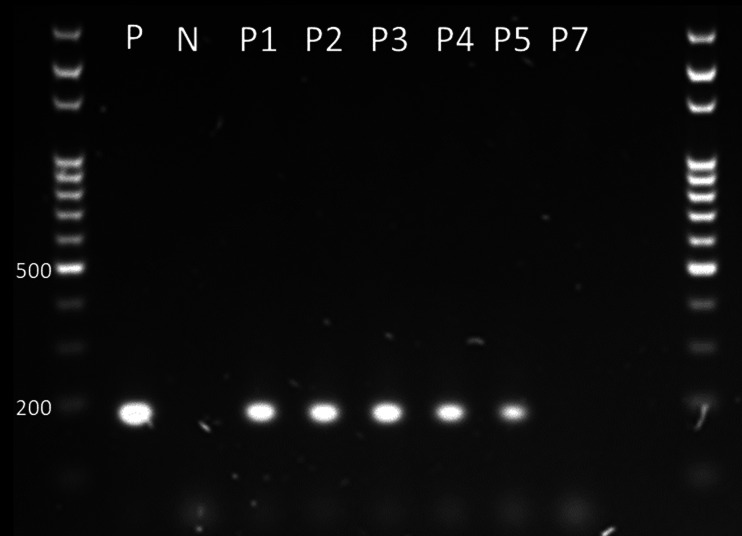
PCR for *erm*(X) in strain 22KM0430 showing plasmid loss after passage 7. P = positive control (DNA used for WGS), first and last lane = 100 bp DNA Ladder (Solis BioDyne), N = negative control, P1-7 = passages. The gel is cropped to the marker range. The original photograph is available as additional file 1

To detect the mechanisms responsible for the observed resistances, screening of the assemblies for resistance genes was performed using ResFinder 4.1 [28] and the Comprehensive Antibiotic Resistance Database (CARD) [29]. An *erm*(X) gene detected by Resfinder was located on the 35.5 kbp plasmid p22KM0430 of the erythromycin- and clindamycin-resistant strain 22KM0430. This plasmid also contained a *tet* gene encoding a Tet(A)/Tet(B)/Tet(C) family tetracycline efflux MFS transporter (which was not detected by Resfinder) but most likely the cause of the tetracycline resistance. Protein BLAST of this gene against the CARD database revealed the highest identity (72%) with Tet(Z) (ARO:3000183) of *Corynebacterium glutamicum* which is below the threshold of gene identity. No antibiotic resistance genes were detected either in the genome or on the plasmid of 21KM1197, which exhibited low MIC levels to all tested antimicrobials (Table 2). A graphical representation of p22KM0430 as compared to p21KM1197 and pNG3, an *erm*(X)-carrying plasmid isolated from *C. diphteriae* isolate ST67 [12] is presented in Fig. 4.

Plasmid p22KM0430 carried three insertion sequences: two of the IS*1380* family and an IS*6-*like element, which was also present with 100% sequence identity on plasmid pNG3. A MobF family relaxase was found on all three of the compared plasmids. Moreover, p21KM1197 displayed a high sequence homology with the section of p22KM0430 outside the insertions (Fig. 4). It was observed that the plasmid was not stably maintained. To document the loss of the *erm*(X)-carrying plasmid, bacteria were passaged on TSA-SB agar up to seven times at 37 °C and 30 °C. For detection of the *erm*(X) gene a PCR was performed using primers ermX_F (5’-TTT TCT CAC CAA CCA CAA GA-3’) and ermX_R (5’-GAG GAG GTT TCT TGT GTG AT-3’) at 0.25µM using FIREPol® Master Mix 12.5mM MgCl_2_ (Solis BioDyne) and the following cycling conditions: 94 °C 3 min [94 °C 30s, 50 °C 30s, 72 °C 30s]x35. The plasmid was no longer detected after seven passages (Fig. 5).

To discover quinolone resistance conferring mutations, nucleotide and amino acid sequences of *gyrA* were aligned and compared using Mega X v. 10.2.02 [30]. Comparison of the *gyrA* sequences between the ciprofloxacin-susceptible strain 21KM1197 and the ciprofloxacin-resistant strain 22KM0430 revealed a G251T transversion leading to an S84I (corresponds to position 83 in *E. coli* numbering) substitution in the quinolone resistance determining region (QRDR) of *gyrA* in 22KM0430 [31]. No mutations were present in *gyrB*.

Antimicrobial susceptibility testing for the *S. canis* isolate from the cystitis case was performed using VITEK®2 AST-ST03 (Biomérieux). The isolate was susceptible to benzylpenicillin, ampicillin, cefotaxime, ceftriaxone, clindamycin, erythromycin, and chloramphenicol.

The corneal ulcer of the Chihuahua harboring the antimicrobial-susceptible strain 21KM1197 was treated with topical serum, moxifloxacin (Vigamox®), oxytetracycline ointment (JENAPHARM®) and a topical lubricant (An-HyPro, an-vision) in combination with systemic treatment with doxycycline (Doxycat®) 5 mg/kg BID and carprofen (Rimadyl®) 4 mg/kg SID. This treatment cleared the ulcer within 4 weeks. The dog suffering from cystitis and harboring antimicrobial-resistant strain 22KM0430 was treated with clindamycin (Antirobe®) orally at a dosage of 5.7 mg/kg twice daily. The clinical signs of polyuria/polydipsia were reportedly alleviated by this treatment even though the strain was resistant. The co-isolated *S. canis* strain however was clindamycin susceptible.

## Discussion and conclusion

The genus *Corynebacterium* contains species which represent important opportunistic pathogens which have been neglected in the past [7, 32]. Paying more attention to this genus and using whole genome sequence analysis for strains remaining unidentified by MALDI-TOF MS can lead to the discovery of novel species in clinical samples. The herein described strains isolated from clinical samples from dogs could not be identified by MALDI-TOF MS and a 16 S rRNA gene sequence based identification placed them within *C. oculi* since the general threshold for species delineation of corynebacteria is around 99% [19]. However, whole genome analyses strongly indicated that these isolates belong to a novel species, which is closely related to *C. oculi*. Especially the low ANI values with *C. oculi* support the placement into a separate species. This is also consistent with the inferred phylogeny based both on the core genome as well as on dDDH. The reliability of these results was based on high quality of the whole genomic sequencing data as indicated by high coverage values, circularization of the genome for both isolates and the 16 S rRNA gene sequences retrieved by Illumina® being 100% identical to the ones generated by Sanger sequencing.

Both samples were retrieved from non-sterile body sites. Thus, the role of the isolates in the clinical conditions of both dogs is unclear and needs to be carefully evaluated. It seems plausible that the strain isolated from the corneal ulcer (21KM1197) was associated with disease as it was isolated from the lesion in pure culture. In the case of cystitis, the role of the isolate is less clear as the urine sample was of spontaneous nature and also contained *S. canis*, which is a known opportunistic pathogen in dogs and a potential cause of cystitis. The *Corynebacterium* sp. being found in greater abundance than the *Streptococcus* might indicate that it played a role in the infection. However, it must be noted that the clinical signs of the dog seemed to improve under treatment with clindamycin, despite the high-level resistance against this drug of the *Corynebacterium* while *S. canis* was susceptible. This suggests that the *Corynebacterium* was either not the primary pathogen or that there was a non-infectious underlying cause for the polyuria and polydipsia of the dog.

Neither animal had conceivably been pretreated with fluoroquinolones, lincosamides, macrolides or tetracyclines. Still, high MIC values of fluoroquinolones, clindamycin, erythromycin and tetracycline were observed in strain 22KM0430. High-range fluoroquinolone resistances were described in ocular isolates of *C. macginleyi* by Eguchi et al. [33]. Several strains were investigated and while most of them showed amino acid substitutions at both positions 83 and 87 (*E. coli*-numbering of the amino acids), one of the isolates showed a single amino acid substitution at position 83. Position 83 in *E. coli*-numbering corresponds to position 84 in our isolates, where the substitution in strain 22KM0430 was found. Whether there were one or two substitutions, almost all of the quinolone-resistant isolates investigated by Eguchi et al. had replaced the original serine with leucine at position 83, whereas our strain had replaced the serine with isoleucine. However, the MIC of ciprofloxacin of 22KM0430 was > 16 mg/L whereas the aforementioned *C. macginleyi* isolate found by Eguchi et al. had a ciprofloxacin MIC of 2 mg/L. Ramos et al. [11] describe two *C. jeikeium* strains with the same substitution and a ciprofloxacin MIC of > 32 mg/L. Therefore, the magnitude of the effect might also be species dependent or driven by an additional yet unknown mechanism.

Lincosamide and macrolide resistance is rather common in human *Corynebacterium* isolates, with *erm*(X) being the most prevalent gene conferring resistance against these antimicrobials [14, 34]. The *erm*(X) gene has been described in various *Corynebacterium* species but the location of the gene varies: while *C. diphtheriae* has been shown to carry the gene on a plasmid as described for strain 22KM0430, in some strains of *C. jeikeium* and *C. striatum, erm*(X) was shown to be located on the chromosome [35]. In *C. xerosis* and other strains of *C. jeikeium*, the gene was found on a transposon (Tn*5432*) which was integrated on a plasmid [35].

The tetracycline resistance observed in strain 22KM0430 was most likely due to the Tet(A)/Tet(B)/Tet(C) family tetracycline efflux MFS transporter encoded on p22KM0430. The same gene was also present on pNG3 indicating possible spread of the associated transposon between species. In a study investigating bacterial isolates from corneal ulcers in dogs from Taiwan, over 50% of the isolates identified as members of the *Corynebacterium* genus were described as being resistant to tetracycline [36]. However, no investigation on the presence of an underlying genetic mechanism was performed.

Roughly half of the plasmid sequence of p22KM0430 was shared by p21KM1197 while the region up- and downstream of the *erm*(X) gene was not present in 21KM1197 suggesting that the *erm*(X) and *tet* gene carrying transposon was integrated into a specific plasmid backbone found in *Corynebacterium* spp.

In conclusion, we described two strains of a *C. oculi* related species obtained from infections in dogs. One strain was isolated from a corneal ulcer indicating a similar predilection site to *C. oculi* in humans. Furthermore, one strain was multi-drug resistant carrying a resistance plasmid, which might be spread to other strains. Clinicians and laboratories should be more aware of corynebacteria as potential pathogens in dogs, and also consider resistance testing prior to therapy.

### Electronic supplementary material

Below is the link to the electronic supplementary material.

Supplementary Material 1: The uncropped gel used for figure 5.

## Data Availability

The datasets generated and analyzed during the current study are available in the GenBank repository under BioProject accession number PRJNA892875.
