# Supplementary figures and images for: Corynebacterium oculi-related bacterium may act as a pathogen and carrier of antimicrobial resistance genes in dogs: a case report
Source: BMC Vet Res. 2023 Nov 29;19:251. doi: 10.1186/s12917-023-03821-y (PMC10763336; doi:10.1186/s12917-023-03821-y)

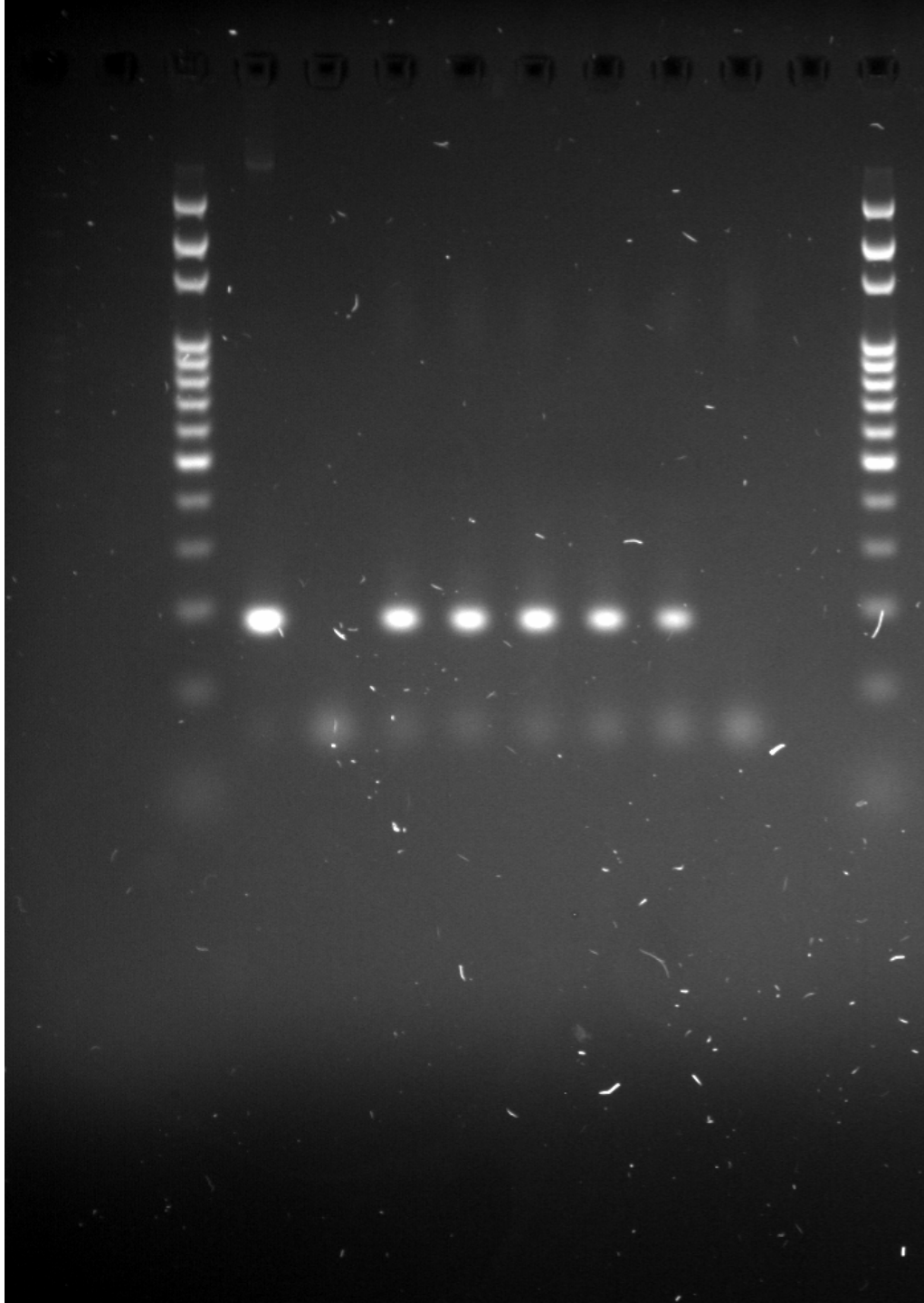

Additional file 1 Showing the uncropped gel used for figure 5.

Supplement: Supplementary file 1 — Supplementary Material 1: The uncropped gel used for figure 5. [file 12917_2023_3821_MOESM1_ESM.pdf]
